# The Role of PPAR*α* Activation in Liver and Muscle

**DOI:** 10.1155/2010/542359

**Published:** 2010-08-18

**Authors:** Lena Burri, G. Hege Thoresen, Rolf K. Berge

**Affiliations:** ^1^Institute of Medicine, Haukeland University Hospital, University of Bergen, 5021 Bergen, Norway; ^2^Department of Pharmaceutical Biosciences, School of Pharmacy, University of Oslo, 0316 Oslo, Norway

## Abstract

PPAR*α* is one of three members of the soluble nuclear receptor family called peroxisome proliferator-activated receptor (PPAR). It is a sensor for changes in levels of fatty acids and their derivatives that responds to ligand binding with PPAR target gene transcription, inasmuch as it can influence physiological homeostasis, including lipid and carbohydrate metabolism in various tissues. In this paper we summarize the involvement of PPAR*α* in the metabolically active tissues liver and skeletal muscle and provide an overview of the risks and benefits of ligand activation of PPAR*α*, with particular consideration to interspecies differences.

## 1. Introduction

Dietary fatty acids (FAs) are not only important for membrane structures and in signalling processes, but also have the ability to influence gene expression by binding to specific transcription factors [1]. One receptor family that acts as mediators to influence transcription according to nutritional state is the peroxisome proliferator-activated receptor (PPAR) family. There are three isoforms of PPAR receptors that have specific, but also overlapping target genes: *α*, *β*/*δ*, and *γ* [2–4]. Early on PPAR activity was thought to mainly influence lipid metabolism, inflammation, and glucose homeostasis. Later it became clear that PPARs also play a role in modulating the processes of cell proliferation and differentiation, apoptosis, and aging [5–8]. The receptors show a nuclear localization in the form of a heterodimer with the retinoid X receptor (RXR). A ligand activated PPAR*α*-RXR heterodimer regulates the transcription of genes by binding to their peroxisome proliferator response elements (PPREs), a process called “transactivation” [9–11]. Besides, a mechanism based on “transrepression” has been described and is reviewed in [12]. The anti-inflammatory actions of PPAR*α* ligands are mostly thought to be based on “transrepression” by the negative interference of PPAR*α* with other transcription factor pathways [13, 14].

Here we focus on the first identified PPAR receptor, PPAR*α* [15], and its activation in different tissues and physiological states in humans and mice. It is expressed at elevated levels in tissues with high metabolic rates, such as the liver, heart, skeletal muscle, kidney, and also in the intestine [12, 16]. Additionally, it is present in cells of the immune system (e.g., macrophages, monocytes, and lymphocytes) [17–19]. The receptor has a central role in fatty acid oxidation, lipid and lipoprotein metabolism, inflammatory responses, and oxidative stress. Its position in the centre of energy balance, lipid metabolism, and inflammation makes it an important factor in the development of obesity-related diseases, and therefore, presents a possible target to influence metabolic disorders. Ligands include saturated and unsaturated FA and their derivatives, hypolipidemic fibrates (ciprofibrate, clofibrate, fenofibrate, and gemfibrozil), and modified fatty acids (e.g., tetradecylthioacetic acid, TTA), as well as xenobiotics [20–22]. In particular during fasting, when free FAs are released into the blood, endogenous lipid-activation is of importance. The importance of PPAR*α* in the cellular metabolic response to fasting was clearly shown in PPAR*α*-null mice [23]. Whereas under normal conditions, these mice do not display a strong phenotype, the absence of PPAR*α* causes lipid accumulation in liver and heart, hypoglycemia, hypothermia, ketonuria, and elevated free fatty acids during fasting ultimately leading to premature death [23]. In contrast, wildtype mice adapt to fasting by induction of hepatic and cardiac PPAR*α* target genes that results in increased FA uptake and oxidation [24].

A great number of animal studies have demonstrated beneficial effects of specific PPAR activation in counteracting metabolic disorders. An increasing number of human studies supports the findings obtained in animal studies. When it comes to PPAR*α* activation, however, it has become clear that not all results obtained in mice can be extrapolated to humans and caution is warranted in predicting tissue-specific effects. 

This paper will focus on the tissues liver and skeletal muscle exploring tissue-specific effects of PPAR*α* activation and stress the differences of human- and mouse-based studies. 

## 2. PPAR*α* in Liver

**Figure 1:**
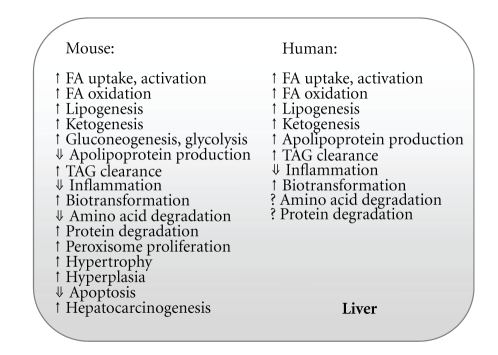
Examples of the multiple metabolic effects of PPAR*α* activation in mouse or human liver. FA, fatty acid; TAG, triacylglycerol.

There are substantial differences between human and mouse target gene expression in terms of the effect of PPAR*α* activation in the liver (Figure 1). Overall, the effect of activation by the PPAR*α* agonist WY14643 is more prominent in mice than in humans [25]. In primary hepatocytes from mice and humans treated with WY14643, only a few target genes were affected similarly in the two species. However, both species share multiple changed gene ontology classes, including lipid metabolism. Individual PPAR*α* regulation was observed for enzymes involved in biotransformation (chemical alterations of compounds in the body), as well as apolipoprotein and bile acid synthesis in human hepatocytes, and glucose homeostasis in mouse hepatocytes [25]. It was proposed earlier that the response might be dampened by quantitative differences of *PPAR*α** expression or different splice forms of *PPAR*α**. Indeed, there exist two splice variants of *PPAR*α** giving rise to an active and inactive receptor in humans [26]. To compare *PPAR*α** expression levels between human and mouse liver is, however, difficult due to daily variations [27] and differing reports have been published. Some reports show lower *PPAR*α** expression levels in human than in rodent liver [28–30], while another shows comparable expression levels between the two species [25].

One of the main pathways involving PPAR*α* regulation in mice and humans includes FA metabolism. In mice, PPAR*α* activation is important for FA metabolism through the induction of genes coding for the fatty acid transporter CD36 [31] and the FA binding protein 1 (FABP1) that brings the FAs from the plasma membrane to the nucleus [32]. Another PPAR*α* target gene is carnitine palmitoyl transferase 1 (*Cpt1*), that codes for a protein important for FA transport into mitochondria [25].Whereas CPT1 is localized to the outer membrane, CPT2, that is also regulated by PPAR*α*, is found in the inner mitochondrial membrane. It converts acyl-carnitine to acyl-CoA and is strongly upregulated by PPAR*α* agonists [33]. Most of the genes of FA metabolism are regulated by PPAR*α* in both humans and mice, however *Cd36* is an example of species-specific induction in mice [25].

Genes encoding for mitochondrial proteins of the *β*-oxidation pathway are induced by PPAR*α* activation, such as acyl-CoA synthetase (*Acs*) coding for an enzyme responsible for activation of FA to their fatty acyl-CoA derivatives. Also genes of the short-, medium-, long- and very-long-chain acyl-CoA dehydrogenases (*Acad -s, -m, -l, -vl*) coding for proteins that catalyze the first step in FA oxidation in a chain length-specific manner, are under the control of PPAR*α*. In addition, the expression of the gene encoding the enzyme acetyl-CoA acyltransferase 2 (ACAA2) involved in the final step of *β*-oxidation, is PPAR*α* dependent. Furthermore, hepatic carnitine synthesis is enhanced by PPAR*α* activation in mice [34, 35]. Carnitine is a conditionally essential nutrient that plays an important role in mitochondrial long-chain FA import for *β*-oxidation [36]. In PPAR*α*-null mice, free carnitine levels were drastically suppressed in plasma and several tissues including liver, the primary site of carnitine biosynthesis. This was consistent with reduced hepatic expression of the genes involved in carnitine biosynthesis (*Bbox1*) and transport (*Octn2*) [37]. In an earlier study, Van Vlies and colleagues established a fasting-induced elevation of these genes that is PPAR*α*-dependent [38]. Both studies point to an essential position for PPAR*α* in carnitine metabolism in mice [37, 38]. No similar indications of PPAR*α*-induced carnitine synthesis have been described in humans. However, pigs that also are a nonproliferative species and are considered similar to humans due to their metabolic features, show an increased carnitine production upon fasting [39]. It is therefore likely that also humans will prove to have a similar response. 

Peroxisomal fatty acid oxidation is important for the partial oxidation of long, very long, and branched FAs. The first characterized PPAR*α* target gene, acyl-CoA oxidase 1 (*Acox1*) encodes the rate-limiting enzyme of this process [40]. After ACOX1 has introduced a double bond to generate enoyl-CoA and H_2_O_2_, the bifunctional protein/enoyl-CoA hydratase (BIEN), that carries two enzymatic activities, performs the second step of *β*-oxidation resulting in 3-ketoacyl-CoA. 3-ketoacyl-CoA is then cleaved by acetyl-CoA acyltransferase 1 (ACAA1) to produce acetyl-CoA [41]. All the above-mentioned genes are under the regulation of PPAR*α* in mice. 

In addition to mitochondrial and peroxisomal *β*-oxidation, *ω*-hydroxylation occurs in smooth endoplasmic reticulum. In both mice and humans, this process is upregulated by the effect of PPAR*α* on expression of cytochrome P450 4A11 (CYP4A11) [25, 42–44]. The hepatic cytochrome P450 4A11 catalyzes *ω*-hydroxylation of medium and long-chain FAs. Subsequently cytosolic dehydrogenases convert them to dicarboxylic acids, which can be further processed by peroxisomal *β*-oxidation. Human PPAR*α* also is a transcriptional regulator of FA oxidation in the different organelles, but shows overlap with mice rather on the pathway than on the gene level [25]. To conclude, PPAR*α* regulates enzymes important for uptake, traffic to final destination, activation, and oxidation of FAs in the three organelles mitochondria, peroxisomes, and microsomes in both mice and humans.

Paradoxically, at the same time as PPAR*α* activation leads to an increase in FA oxidation, it also augments FA synthesis by affecting gene expression levels of several enzymes involved in lipogenesis. In mice, PPAR*α* stimulates the conversion of malate into pyruvate to generate NADPH for lipogenesis by upregulating the expression of malic enzyme (ME1) [45]. Besides, the ∆5, ∆6, and ∆9 desaturases, rate-limiting enzymes in the synthesis of polyunsaturated FAs (PUFAs) from saturated FAs, are found in increased amounts after PPAR*α* activation [46–48]. The induction of desaturases could help to ensure that there are always enough PUFAs for their diverse functions, including being effective PPAR*α* agonists as proposed by others [46]. Likewise, PPAR*α* activation in human hepatocytes induces the expression of several target genes involved in FA synthesis [25]. 

Other crucial processes requiring PPAR*α* activation are lipoprotein synthesis and assembly. The impact of PPAR*α* agonist on lipoprotein gene expression in humans or mice is distinct. The use of fibrates in humans leads to reduced plasma triacylglycerol (TAG) levels and increased high-density lipoprotein (HDL) cholesterol levels. In mice, plasma TAG as well as HDL levels are lowered. The liver, besides the intestine, determines the amount of HDL in plasma by regulating HDL synthesis and catabolism. The reason for the species-specific opposite effect of PPAR*α* activation on HDL levels is probably increased production levels of apolipoprotein A-I (APOA1) and APOA2 in humans [49, 50] and suppressed (APOA1) or unchanged (APOA2) expression in mice [51]. These apolipoproteins are part of HDL cholesterol and are crucial for reverse cholesterol transport from peripheral cells to the liver, where excess cholesterol can be eliminated into the bile [52]. The liver is also the place where very low-density lipoprotein (VLDL) particles are assembled and then secreted into the plasma. The VLDL amount in peripheral cells is influenced by lipoprotein lipase (LPL). The hepatic expression of this hydrolase, which mediates VLDL triglyceride lipolysis, is upregulated by PPAR*α* [53]. Moreover, its activity is stimulated by APOA5 and inhibited by APOC3. Activation of PPAR*α* increases *APOA5* [54–56] and decreases *APOC3* [57] transcription, resulting in a plasma TAG lowering effect, thereby, together with increased HDL concentrations, reducing the risk for atherosclerosis in humans [58]. 

The removal of excess cholesterol from the body is via the bile, a fluid produced in the liver, stored in the gall bladder, and secreted into the small intestine. Cholesterol is eliminated either intact or as bile acids that are steroid acids made from cholesterol. In humans, the two main bile acids synthesized in the liver, are chenodeoxycholic acid (CDCA) and cholic acid (CA) [59, 60]. Due to their amphipathic character they aid in the small intestine for the digestion and absorption of dietary lipids. There is controversy in the literature regarding the regulation of the rate-limiting enzyme in hepatic bile acid synthesis, called cholesterol 7*α*-hydroxylase (CYP7A1). Some reports suggest a transcriptional upregulation of *Cyp7a1* upon PPAR*α* activation in mice [61, 62]. In particular, the upregulation of *Cyp7a1* under fasting conditions and the downregulation of this enzyme in PPAR*α*-null mice corroborate a PPAR*α* regulatory involvement and suggest increased expression upon fasting-induced PPAR*α* activation [62]. Other studies support a downregulation of this endoplasmic reticulum enzyme upon induction with PPAR*α* agonists in both humans and rodents [63–67]. This could be a potential risk for gallstone formation, if in humans receiving treatment with fibrates, bile acid synthesis is decreased over a longer period of time by a hepatic decrease of CYP7A1 activity. On the other hand, gene expression of sterol 12*α*-hydroxylase (*Cyp8b1*), an enzyme involved in CA synthesis, is increased under fasting and also with ligand-induced PPAR*α*-activation in both rodents and humans [62, 67, 68]. This protein of the cytochrome P450 family controls the balance between CA and CDCA levels. Upon *Cyp8b1* induction, higher CA concentrations positively influence the bile acid composition by increasing cholesterol solubility. 

Important under conditions of extended fasting is the process called ketogenesis. In mice and humans, the production of ketone bodies is under the control of PPAR*α* that upregulates the gene expression of mitochondrial 3-hydroxy-3-methylglutaryl-CoA synthase (*Hmgcs2*), coding for the rate-limiting enzyme of ketogenesis [25, 69, 70]. Of particular importance in regulating ketogenesis, in addition to FA oxidation, TAG clearance, and de novo lipogenesis is the ‘hormone-like' fibroblast growth factor 21 (FGF21) [71–73]. Its hepatic expression is PPAR*α*-dependent and is induced by fasting, a ketogenic diet, and WY14643 [25, 71, 74, 75]. FGF21 positively influences lipid and glucose metabolism, in addition to insulin sensitivity in animals [76]. 

Hepatic gluconeogenesis is also regulated during fasting, when the liver changes from glucose uptake and glycogen synthesis to glucose production. The chain of reactions converting glycerol, lactate, or glucogenic amino acids to glucose involves the two rate-limiting enzymes, phosphoenol-pyruvate carboxykinase (PEPCK) and pyruvate carboxylase (PYC). Of these two genes, only the promoter for *Pepck* was found to have a functional PPRE in mice [77]. The induction of other enzymes in this pathway is PPAR*α*-dependent, such as glycerol-3-phosphate dehydrogenase (GPDH) and glycerol kinase (GK), as well as the aquaporins (AQP) 3 and 9 that act as liver glycerol import channels [78]. The observation that PPAR*α*-null mice manifest lower fed and fasted glucose levels supports an involvement of PPAR*α* in hepatic glucose production [77]. However, another report proposes as a reason for fasting hypoglycemia, the preferential channelling of glucose-6-phosphate to hepatic glycogen stores and shows unchanged glucose 6-phosphate synthesis in PPAR*α*-null mice [79]. The pathway glycolysis/gluconeogenesis is specifically affected by PPAR*α* activation in mice and shows no response in human primary hepatocytes [25].

The enzyme glyoxylate reductase/hydroxypyruvate reductase (GRHPR) is important in the channelling of carbons from the glyoxylate cycle into gluconeogenesis or into the urea cycle depending on the body energy demands. In mice, PPAR*α* activation (e.g., in the fasted state) is crucial in inducing transcriptional activation of *Grhpr*, thereby favouring a conversion of hydroxypyruvate to D-glycerate, a substrate needed in glucose synthesis [80]. In humans however, *GRHPR* expression was shown to be PPAR*α*-independent due to promoter reorganisation during primate evolution. Moreover, alanine:glyoxylate aminotransferase (AGT), an enzyme of the glyoxylate cycle with two enzymatic activities is positively regulated by PPAR*α* [80]. Its transaminase activity leads to the production of glycine and hydroxypyruvate. 

Beyond the transcriptional activation of genes involved in lipid and glucose metabolism, the PPAR*α* agonist WY14643 affects amino acid metabolism in rodents [81, 82]. The metabolic consequences include alterations in plasma amino acid levels. Whereas branched-chain amino acid amounts showed no change upon PPAR*α* activation with WY14643, a significant increase in various glucogenic and some ketogenic amino acids was detected in rats [82]. Only one amino acid was lowered, namely arginine, a conditionally nonessential amino acid made in the urea cycle. mRNA levels of enzymes involved in the conversion of citrulline to arginine in the kidney are unknown, but hepatic levels of argininosuccinate synthetase (*Ass*) and argininosuccinate lyase (*Asl*) show a decrease [81, 82]. The exact mechanism of PPAR*α* regulation of amino acid metabolism is unknown but certain genes involved in the regulation of amino acid degradation have also been shown to be negatively regulated, with the exclusion of *Grhpr* and arginase (*Arg1*) [81, 82]. The decreased amino acid degradation upon WY14643 treatment is accompanied by an increase in protein degradation. Some possible explanations for the observed amino acid mobilization upon PPAR*α* induction are give in [82] and might be due to increased hepatic growth. The current findings are restricted to rodents and it is unclear at present if the situation is similar in humans that show no liver enlargement. One study points to a different situation in humans and describes increased plasma arginine levels after fenofibrate treatment of hypertriglyceridemic men [83]. The findings in rodents are limited to WY14643 treatment and it remains to be shown if they are of general character for PPAR*α* ligands. The clofibrate-induced increased oxidation of branched-chain amino acids seems to be due to its direct inhibitory actions on branched-chain *α*-keto acid dehydrogenase kinase (BCKDK) that regulates the key enzyme of this process, and not due to effects mediated through PPAR*α* activation [84].

Additionally, in mice, PPAR*α* activation inhibits inflammatory gene expression by downregulation of acute phase proteins such as C-reactive protein (CRP), fibrinogen, and serum amyloid A (SAA) resulting in reduced hepatic inflammation and risk for cardiovascular disease and cancer [85]. Likewise in humans, there is a similar downregulation of plasma acute phase proteins after fenofibrate treatment [86]. Recently, it was demonstrated that the expression of the transcription factor CREBH that is exclusively found in the liver, is regulated by PPAR*α* in both mice and humans [25]. It plays an important role in the activation of the acute inflammatory response and is also a regulator of hepatic gluconeogenesis [87, 88]. 

Described in mice is the reduced risk of liver damage by chemical-induced stress. Exposure to hepatotoxic agents like the environmental pollutant carbon tetrachloride (CCl_4_) induces reversible liver damage [89]. The underlying reason is a decreased resistance to oxidative stress that leads to lipid peroxidation, altered calcium homeostasis, and membrane damage. Stimulated mRNA expression of uncoupling protein 2 (*Ucp2*) by PPAR*α* in rodents results in uncoupling of the proton gradient across the inner mitochondrial membrane and a downregulation of reactive oxygen species (ROS) induced by CCl_4_ metabolites [90, 91]. In addition, PPAR*α* helps to protect from chemical-induced oxidative stress by upregulating genes of the chaperone family and of the proteasome, thereby influencing protein folding and degradation of harmed proteins in mice [92]. Furthermore, the observation that PPAR*α*-null mice demonstrate decreased longevity, where stress response genes are of importance, and that PPAR*α* expression decreases with age, suggests an involvement of PPAR*α* in this process [7].

In rodents, long-term administration of PPAR*α* leads to increased peroxisome proliferation, in addition to hepatic hypertrophy and hyperplasia that will ultimately result in liver tumors [93–98]. The carcinogenic response is based on enhanced cell replication that might increase the risk for DNA damage and altered oncogene and tumor suppressor gene expressions. Moreover, there is evidence for suppressed apoptosis in liver cells, a process important for the removal of damaged cells [99–102]. There is also a close relationship of PPAR*α*-induced cancer formation with increased production of ROS due to peroxisome proliferation that might contribute to DNA damage [103]. 

Shah and colleagues have proposed changed hepatic microRNA (miR) expression via PPAR*α*-regulation as the reason for liver cancer formation [104]. miRs are 21–23 nucleotide long sequences that are suggested to regulate the expression of up to 30% of all genes [105, 106]. Experimental evidence pointed to PPAR*α*-involvement in several changed miR levels, in particular in the downregulation of miR let-7c by an as yet unidentified mechanism [104]. Let-7c controls c-Myc protein levels, a transcription factor regulating target genes involved in cell proliferation. Downregulation of let-7c stabilizes *c-Myc* mRNA leading to the expression of c-Myc target genes. This could be a reason for enhanced hepatocyte proliferation, that together with the induction of oxidative stress might lead to hepatocarcinogenesis in rodents. Induction of hepatocarcinogenesis seems to be restricted to rodents and is not documented in humans (extensively reviewed in [107]). Cancer formation after PPAR*α* activation in tissues other than the liver has been described in rats and includes testicular (Ledig cell) and pancreatic acinar cell tumors [108]. However, if these findings are of significance for humans requires further in-depth risk assessments.

In summary, the hepatic response to PPAR*α* activation is essential under fasting conditions. PPAR*α* activation by FAs released from the adipose tissue leads to induction of several metabolic processes in mice: *β*-oxidation, ketogenesis, glycolysis/gluconeogenesis, with concomitant reduction of amino acid catabolism and an anti-inflammatory response. The changes result in an increased plasma concentration of glucose and ketone bodies and decreased urea and acute phase proteins. PPAR*α* is important in both mice and humans for the regulation of lipid metabolism. In contrast to mice, humans show no effect on the glycolysis/gluconeogenesis pathway. One pathway specifically affected in humans and not in mice is apolipoprotein production. In humans treated with a PPAR*α* activator, hepatic transcription activation leads to decreased VLDL production and plasma TAG levels, but increased HDL cholesterol, important parameters in the treatment for dyslipidemia, type 2 diabetes, or cardiometabolic disorders. 

## 3. PPAR*α* in Skeletal Muscle

In human skeletal muscles, three main muscle fiber types, type I (oxidative, slow twitch), IIA (intermediate) and IIX (glycolytic, fast twitch), can be delineated based on histochemical, functional and biochemical properties (reviewed in [109]). In human skeletal muscle cells *in vitro*, *PPAR*α** was shown to be induced early during myocyte differentiation [110, 111]. A correlation between the expression of *PPAR*α**, proportion of type I fibers and endurance exercise has been found in human skeletal muscle *in vivo* [112, 113]. The expression of *PPAR*α** (as well as of *PPAR*δ** and the *PPAR*γ** coactivator (*PGC)- *1*α* and *-1ß*) in skeletal muscle was increased in athletes and reduced in spinal cord-injured subjects [113]. The observed increase of *PPAR*α** expression after endurance training [112, 114] was greater in type I fibers than in type IIA and IIX fibers [112]. Also in rat skeletal muscle, fiber-type specific PPAR*α* activation was found. When treated with the PPAR*α* agonist fenofibrate, 26 genes were identified that were significantly regulated in soleus (type I) but not in quadriceps femoris (type II) rat muscle [115]. The correlation of *PPAR*α** expression and exercise has not been found in animal studies. In rats, four weeks of exercise did not change the *PPAR*α** mRNA expression in skeletal muscle in control chow-fed animals, and in fat-fed rats exercise counteracted the diet-induced increase of *PPAR*α**expression [116]. 

Both in human and rodent skeletal muscle, activation of PPAR*α* affects lipid metabolism. Activation of PPAR*α* by a potent agonist (GW7647) in differentiated human myotubes* in vitro* stimulated lipid oxidation [110, 117] and decreased accumulation of TAG [110]. Other, less potent PPAR*α* agonists did not increase lipid oxidation in human myotubes [118]. In the same cell model, GW7647 upregulated the expression of pyruvate dehydrogenase kinase (*PDK*)4 [119]. PDK4 is an important isoenzyme regulating the activity of pyruvate dehydrogenase complex. The enzyme phosphorylates and inhibits the pyruvate dehydrogenase complex and thereby blocks the entry of carbohydrates into the mitochondria for oxidation (for reviews see [120, 121]. *Pdk4* was also induced in rat gastrocnemius muscle after treatment of the animals with the PPAR*α* agonist WY14643, by streptozotocin-induced diabetes, or by starvation, i.e. conditions where increased levels of long-chain fatty acids may activate PPAR*α* [122]. Pathway analysis of the genes significantly regulated in soleus (type I), but not in quadriceps femoris (type II) muscle by fenofibrate in rats, revealed that the most significant function represented in the gene set was lipid metabolism [115]. Treatment with a potent PPAR*α* agonist increased the expression of *Cpt-1* in hamster soleus muscle [123]. 

Influence of PPAR*α* on both lipid and glucose metabolism was highlighted in transgenic mice overexpressing PPAR*α* in skeletal muscle [124]. In these animals many known PPAR*α* target genes involved in cellular fatty acid import and binding, TAG synthesis, and mitochondrial and peroxisomal *β*-oxidation were activated, and genes involved in cellular glucose utilization were downregulated in skeletal muscle. Basal and insulin-stimulated glucose uptake was reduced in isolated skeletal muscle, and the transgenic animals developed glucose intolerance despite being protected from diet-induced obesity [124]. In contrast, in PPAR*α*-null mice, glucose tolerance, insulin-stimulated glucose disposal and glucose uptake were increased in spite of high fat-induced weight gain and increased levels of TAGs in muscle [124]. In another study, fatty acid oxidation in skeletal muscle was found to be reduced by 28% in starved PPAR*α*-null mice compared to wild type (WT) mice, however in fed animals fatty acid oxidation in PPAR*α*-null and WT mice was similar [125]. TCA cycle intermediates, amino acids and short-chain acylcarnitine species were reduced in skeletal muscle of PPAR*α*-null mice compared to WT mice, indicating impaired TCA cycle flux and increased protein catabolism combined with defects in fatty acid catabolism in PPAR*α*-null mice [37].

In humans and mice, a negative side effect of PPAR*α* activation in muscle is in rare cases (<1%) muscle weakness and pain (myopathy) or very seldom breakdown of muscle (rhabdomyolysis) [126–129]. In particular, type I fibers are affected by skeletal muscle toxicity in rats [115]. The exact mechanisms are unclear at present, but might include oxidative stress and tissue damage from elevated peroxisomal and mitochondrial *β*-oxidation [130].

PPAR*α* also seems to exert a role in protecting against ischemic injury in skeletal muscle as well as in heart and liver [131]. Thus, in mouse skeletal muscle, loss of the oxygen sensor prolyl oxidase (PHD)1 was found to lower oxygen consumption by shifting to a more anaerobic glucose utilization through activation of PPAR*α*-dependent genes [131].

**Figure 2:**
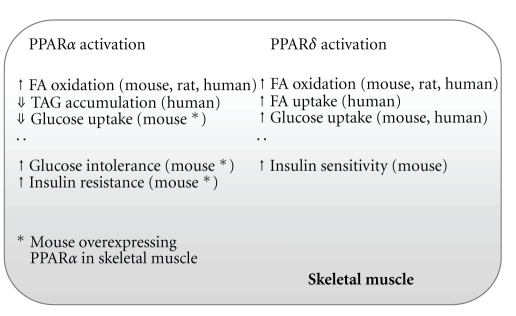
Examples of metabolic effects of PPAR*α* or PPAR*δ* activation in skeletal muscle. FA, fatty acid; TAG, triacylglycerol. For references, see the text.

Another PPAR, PPAR*δ*, is the most abundant PPAR isoform in skeletal muscle. Similar to *PPAR*α**, the expression of *PPAR*δ** has been described to be higher in type I fibers compared to type II fibers (reviewed in [132]). Also alike to PPAR*α*, activation of PPAR*δ* induces a number of genes involved in fatty acid import and oxidation, and increases lipid oxidation in skeletal muscle [125, 133–136], indicating redundancy in the functions of PPAR*α* and *δ* as regulators of fatty acid metabolism [125]. However, in contrast to PPAR*α*, activation of PPAR*δ* has been shown to increase glucose uptake [136, 137] and prevent insulin resistance in skeletal muscle (Figure 2) [138].

In summary, PPAR*α* has been shown to be involved in lipid and glucose metabolism in skeletal muscle. PPAR*α* activation increases lipid oxidation and decreases TAG accumulation. Overexpression of *PPAR*α** in skeletal muscle causes reduced glucose uptake in muscle and glucose intolerance in the animals, while PPAR*α*-null mice show increased glucose tolerance, increased insulin-stimulated glucose disposal and enhanced glucose uptake in skeletal muscle, in spite of high fat-induced weight gain and increased levels of TAGs in muscle. Thus, PPAR*α* activation may potentially exert both beneficial and undesirable effects on skeletal muscle fuel metabolism. Activation of PPAR*α* and PPAR*δ* seems to have overlapping effects on fatty acid metabolism, but possibly different effects on glucose metabolism in skeletal muscle.

## 4. Concluding Remarks

The transcription factor PPAR*α* influences metabolism through activation of many target genes in a variety of metabolically active tissues, in particular under fasting conditions. Cross-species prognostics are not always possible due to differences in metabolism, expression levels, or diet. While observations in rodents could have pointed to risks for human treatment with PPAR*α* agonists (e.g., hepatocarcinogenesis, skeletal muscle insulin resistance, and myopathy) it has been shown that in humans, PPAR*α* activation is a useful therapeutic target in treating metabolic disorders. Clinical studies on drug-induced PPAR*α* activation include fibrates, statins, and more recently the combination of statins with fibrates. In humans, fibrates have the characteristic of reducing TAG levels and increasing HDL cholesterol, however not all trials show a vascular benefit. In some trials, clinical end-points like the rate of coronary heart disease in type 2 diabetes patients (VAHIT: Veterans affairs HDL intervention Trial, [139]) or the progression of atherosclerosis in young men after a first myocardial infarction (BECAIT: Bezafibrate Coronary Atherosclerosis Intervention Trial, [140]) could be reduced by treatment. Statin therapy shows more consistent benefits with decreased plasma LDL cholesterol levels and reduced vascular disorders and death [141]. The Action to Control Cardiovascular Risk in Diabetes (ACCORD) lipid study, addressed whether a fibrate (fenofibrate) and statin (simvastatin) combination would reduce the rate of cardiovascular events more than individual treatments in type 2 diabetes patients [142]. The combination treatment however did not influence the primary outcome significantly more than simvastatin alone, but instead showed a sex-dependent difference, with more benefits for men than women. 

Rodent studies are mostly done in male animals, but the response of PPAR*α* activation in male versus females was investigated in some studies and seems to be influenced by estrogen [143, 144]. This female hormone inhibits PPAR*α* action and represses lipid regulatory pathways in the liver. Thus, in the treatment with PPAR*α* agonists, gender-differences have to be taken into consideration and while therapy might be advantageous against lipid disorders in men and postmenopausal women with no interfering estrogen, premenopausal women might not benefit from the same treatment [145].
